# Recombinant CXCL16 reduces brain injury by modulating microglial phenotype and attenuating apoptosis in acute ischemic stroke

**DOI:** 10.1038/s41598-026-66143-7

**Published:** 2026-08-22

**Authors:** Hongyi Sun, Cheng Zhou, Jing Hu, Tengfei Luan, Taoli Lu

**Affiliations:** 1 Center for Neurological Function Test and Neuromodulation, West China Hospital, West China Xiamen Hospital, Sichuan University, 699 Jinyuan West Road, Xingbin Street, Jimei District, Xiamen, 361000 China; 2 Department of Critical Care Medicine (ICU), The First People’s Hospital of Yibin, No. 65 Wenxing Street, Cuiping District, Yibin, 644000 Sichuan China; 3 Department of Clinical Psychology, Zhongshan Third People’s Hospital, 80 Tianbian Zheng Street, Nanlang Town, Zhongshan, 528400 China; 4 Department of Neurology, West China School of Medicine, Sichuan University, Sichuan University affiliated Chengdu Second People’s Hospital, Chengdu Second People’s Hospital, Chengdu, 610017 Sichuan China; 5 Department of Neurosurgery, The First Hospital of Jilin University, Changchun, Jilin China; 6 Department of Neurology, University of Giessen, Giessen, Germany

**Keywords:** CXCL16, Microglia, Ischemic stroke, Apoptosis, Immunology, Neurology, Neuroscience

## Abstract

Chemokines are traditionally known for their roles in immune cell recruitment during inflammation, but emerging evidence suggests that they may also directly regulate cellular states within the central nervous system. Specifically, it remains unclear whether CXCL16 affects microglial functional states in ischemic stroke. Here, we demonstrated that recombinant CXCL16 (rCXCL16) modulated the expression of inflammation- and repair-associated markers in primary microglia and in the ischemic brain. Functionally, microglia pretreated with rCXCL16 increased HT-22 cell viability and reduced apoptosis in an indirect co-culture system. Consistently, in vivo administration of rCXCL16 reduced infarct size, restored neurobehavior performance, and suppressed apoptosis in experimental stroke in mice. These findings identify rCXCL16 as a modulator of microglial responses and suggest that its neuroprotective effects are associated with reduced inflammatory marker expression and attenuation of apoptotic injury after ischemic stroke.

**Supplementary Information:**

The online version contains supplementary material available at 10.1038/s41598-026-66143-7.

## Introduction

Ischemic stroke represents as a major contributor to global burden of death and long-term disability, representing approximately 80% of all stroke cases^1–3^. The reperfusion strategies, including intravenous thrombolysis and mechanical thrombectomy, have improved patients’ quality of life, but their overall clinical benefit is still constrained by narrow therapeutic windows, restricted availability of specialized care, and procedure-related complications^4,5^. Consequently, a substantial proportion of patients continue to experience lasting neurological deficits, underscoring the need to develop novel therapeutic strategies to enhance functional recovery.

Chemokines are regulators of post-ischemic neuroinflammation, mediating the recruitment of peripheral immune cells into the central nervous system^6,7^. Among them, CXCL16 has attracted increasing attention due to its involvement in neuroinflammatory regulation and its interaction with microglia^4,8–10^. Microglia exhibit marked temporal and functional heterogeneity following ischemic stroke and adopt a continuum of context-dependent states rather than discrete M1/M2 phenotypes. These states are characterized by overlapping inflammatory, homeostatic, phagocytic, and repair-associated programs that evolve across different stages of injury and recovery. However, the role of CXCL16 in shaping microglial functional states after ischemic injury remains poorly understood.

Apoptosis dominates post-ischemic cell death^11,12^. Unlike necrotic cell death, apoptosis is a highly regulated process in the ischemic penumbra, making it an attractive therapeutic target for limiting secondary brain injury after stroke^13,14^. Given that CXCL16 has been shown to exert neuroprotective effects under pathological conditions^15^, it is of interest to investigate whether CXCL16 may attenuate apoptotic cell death in the context of ischemic injury.

These observations prompted us to determine the involvement of CXCL16 on microglial functional states under cerebral ischemic conditions and to explore its potential role in modulating apoptotic cell death.

## Materials and methods

### Animals

Male C57BL/6J mice, 8 weeks old and weighing 21–25 g, were purchased from Vital River (Beijing, China). All animals were acclimatized for one week in an SPF-grade facility with controlled conditions (24 ± 2 °C, 40 ± 5% humidity). Following random assignment to the experimental or control group, all animals were maintained on a 12-hour light/dark cycle. Mice were euthanized by CO_2_ inhalation in an induction chamber with a displacement rate of 30% chamber volume/min. Following respiratory arrest, animals were maintained in the chamber for at least 2 min, and death was subsequently confirmed by cervical dislocation. All experimental procedures were performed in accordance with the Guide for Care and Use of Laboratory Animals and the Chinese national standard GB/T 35,892 − 2018. The study was approved by Institutional Animal Care and Use Committee of Shenzhen Rongwan Biomedical Experimental Animal Center.

### Primary microglia culture

Primary microglia were isolated from neonatal C57BL/6J mice at postnatal days 0–3. Pups were not selected or stratified according to sex, and cerebral cortices from three pups within the same litter were pooled for each culture flask. The cells were then maintained in DMEM with 10% FBS and 1% penicillin-streptomycin (P/S) under standard conditions. The cerebral cortices were retained and mechanically dissociated by gentle trituration with a 10 mL pipette. The cell suspension was transferred to T75 culture flasks with three brains per flask. We replaced the culture medium after 24 h and every 5 days thereafter. After 14 days in vitro, the flasks were placed on an orbital shaker and incubated overnight to detach microglia. The next day, the supernatant with primary microglia was gathered and spun at 200 g for 5 min. The cell pellet was then redissolved, and cells were counted and seeded as required for subsequent experiments.

### HT-22 cell culture

HT-22 cells were maintained in DMEM supplemented with 10% FBS. Every 1–2 days, the culture medium was refreshed.

### Oxygen-glucose deprivation (OGD) model

HT-22 cells were cultured to 80–90% confluence. Primary microglia were subjected to OGD 1–2 days after seeding. Cells were washed twice with DPBS, and the culture medium was replaced with glucose-free DMEM. The cells were then exposed to hypoxic conditions (1.5% O₂) for the indicated durations. An oxygen concentration of 1.5% was selected based on published studies using organotypic hippocampal slice OGD and primary microglial hypoxia models^16,17^. The duration of OGD was subsequently optimized in primary microglia. After OGD, the cells were washed with DPBS and returned to their respective culture media. Reoxygenation was performed under normoxic conditions at 37 °C with 5% CO₂ for 24 h. The cells were subsequently harvested for further analyses.

### In vitro rCXCL16 treatment

Before treatment, primary microglia were deprived of serum in DMEM with 1% P/S for 2 h. For experiments under hypoxic conditions, cells were treated with increasing amounts of rCXCL16 (30, 60 and 120 ng/ml) as indicated and incubated overnight prior to OGD/R induction. For experiments involving LPS stimulation, microglia were co-treated with 120 ng/ml CXCL16 and 500 ng/ml LPS.

### Co-culture system of primary microglia and HT-22 cells

Microglia were seeded into 0.4 μm transwell inserts, and neurons were maintained in the lower wells. Both cell types underwent OGD/R modeling following the same protocol as described in the drug treatment section. The timing was coordinated such that microglia completed reoxygenation just as neurons began reoxygenation. At this point, transwell inserts containing microglia were transferred to the neuronal wells and co-cultured for 24 h.

### 3-(4,5-dimethylthiazol-2-yl)−2,5-diphenyltetrazolium bromide (MTT) assay

Cell viability was assessed using the MTT assay. Active cells reduce tetrazolium salts to purple formazan crystals, forming the basis of this assay. Cells were exposed to 100 µl of MTT solution (5 mg/ml in PBS) at 37 °C for 4 h following OGD/R. The resulting crystals were solubilized in 1 ml of DMSO with gentle shaking for 5 min. Subsequently, a 200 µL portion of the dissolved solution from each well was moved to a 96-well microplate for spectrophotometric measurement of absorbance at 570 nm.

### 2,3,5-triphenyltetrazolium chloride (TTC) staining

The brains of mice were rapidly extracted and placed in a brain matrix 24 h after MCAO, following euthanasia. The brains were coronally sectioned into 2 mm-thick slices. Afterward, the sections were immersed in a 2% TTC solution and kept at 37 °C for 15 min. After staining, the slices were imaged, and the volume of the infarct was measured with ImageJ software. To correct for brain edema, infarct volume was determined using the indirect method: infarct volume = contralateral hemisphere volume − non-infarcted ipsilateral hemisphere volume.

### Middle cerebral artery occlusion (MCAO) model establishment and drug administration

We fasted the mice before surgery but allowed them free access to water. We induced and maintained anesthesia with 1.5% isoflurane and maintained body temperature at 37 °C using a heating pad. Laser Doppler flowmetry with a flexible probe placed on the skull overlying the MCA territory monitored cerebral blood flow. After cervical disinfection, a midline skin incision was made to dissect the left carotid bifurcation. The distal common carotid artery (CCA) and the external carotid artery (ECA) were ligated. The internal carotid artery (ICA) was temporarily occluded with an arterial clip. To induce MCAO, we made a small incision in the CCA, inserted the filament (Doccol Corporation, USA), and advanced it into the ICA until resistance was detected. Forty-five minutes later, the filament was withdrawn, and the CCA was ligated at the incision site. After each use, the filaments were cleaned, sterilized, and stored in sterile saline at 4 °C. We performed all surgical procedures on the sham group mice except that the filament was not inserted.

Recombinant mouse CXCL16 (Peprotech) was solubilized in saline and delivered via intracerebroventricular (ICV) injection at 1 h post MCAO. For dose-response studies, mice received 10, 30, or 60 µg/kg of CXCL16 in 2 µL of saline. Stereotaxic frame placement (RWD Life Science, Shenzhen, China) was performed under anesthesia for each animal, and the injection was targeted to the left lateral ventricle (3 mm below the dura, 1 mm lateral to bregma). The solution was infused at a constant rate of 0.2 µL/min using a R462 high-precision microinjection pump (RWD Life Science, Shenzhen, China).

### Modified neurological severity Score(mNSS)

mNSS was used to evaluate motor coordination, sensory reflexes, and postural stability. The scoring system ranges from 0 to 18, and higher scores correlate with more severe neurological dysfunction.

### TUNEL staining

TUNEL staining was carried out using a One-step TUNEL In Situ Apoptosis Kit (Elabscience, Wuhan, China).

### Immunofluorescence staining

After transcardial perfusion with ice-cold PBS and 4% paraformaldehyde (PFA), brains were collected and fixed overnight in 4% PFA at 4 °C. Tissues were dehydrated in a 30% sucrose solution until they subsided; this process was repeated once with fresh 30% sucrose. Primary microglia were seeded on coverslips, fixed with 4% PFA for 10 min, permeabilized with 0.25% Triton X-100 for 5 min, and blocked with 5% bovine serum albumin (BSA) at RT for 30 min. Brain sections and cultured cells were then incubated overnight at 4 °C in a humidified chamber with primary antibodies against Iba-1 (1:200, Oasis Biofarm), CXCR6 (1:200, Abclonal), CD86 (1:200, Affinity Biosciences), and CD206 (1:400, CST). After three washes with PBS, samples were incubated with the corresponding secondary antibodies for 1 h at room temperature in the dark. Nuclei were counterstained with DAPI. Coverslips and sections were mounted using anti-fade mounting medium and stored at 4 °C. Images were obtained using a Zeiss LSM900 confocal microscope (Carl Zeiss, Germany).

For quantitative analysis, brain sections from six mice per group (*n* = 6) were examined. For each mouse, three coronal sections were selected, and five randomly chosen fields per section were imaged. Quantification was performed in a blinded manner, and data from multiple fields and sections were averaged to obtain a single value per animal.

### Western blotting

Total protein was extracted and quantified as described previously^18^. Electrophoresis was run at 80 V for 30 min, and then at 120 V until the dye front reached the bottom of the gel. Before transfer, the PVDF membrane was activated by immersing it in methanol for 3 s. Protein transfer was performed by semi-dry blotting at 1.0 A for 30 min. A blocking step was performed using TBST containing 5% BSA for 1 h. The membranes were then incubated overnight at 4 °C with the following primary antibodies: anti-CXCL16 (1:1000, Affinity Biosciences), anti-CXCR6 (1:1000, Affinity Biosciences), anti-Bax (1:1000, Proteintech), anti-Bcl-2 (1:1000, Proteintech), anti-Arg-1 (1:1000, HUABIO), anti-iNOS (1:1000, HUABIO), and anti-β-actin (1:5000, Proteintech). After washing the next day, the membranes were incubated with a 1:5,000 dilution of secondary antibody for 1 h at RT. After washing, signal detection was performed by incubating the membrane with ECL substrate, followed by imaging using the ChemiDoc imaging system (Bio-Rad, USA). Band intensities were quantified using ImageJ software.

### Reverse transcription quantitative PCR (RT-qPCR)

Total RNA was extracted from primary microglia using the Universal RNA Extraction Kit (Accurate Biotechnology, Hunan, China). The concentration and purity of the extracted RNA were determined using a Metash B-600 ultra-micro spectrophotometer (Metash, Shanghai, China). cDNA was synthesized using the RT Mix Kit with gDNA Clean for qPCR (Accurate Biotechnology, Hunan, China). qPCR was carried out using the SYBR Green Premix Kit (Accurate Biotechnology, Hunan, China) on a LightCycler 480 II Real-Time PCR System (Roche, Switzerland). Primer sequences are listed in Supplementary Table 1. We normalized target gene expression to β-actin and calculated relative levels using the 2⁻ΔΔCt method.

### Flow cytometry

Cell apoptosis was evaluated using a FITC-labeled Annexin V and PI Apoptosis Detection Kit (Sangon Biotech, Shanghai, China). A suspension of 1 × 10⁶ cells was incubated with Annexin V-FITC and PI for 15 min in the dark. Subsequent analysis was performed on a BD FACSCalibur flow cytometer. Early and late apoptotic cells were quantified and expressed as a percentage of total cells.

### Single-cell RNA-sequencing analysis

Raw count matrices from the GSE227651 dataset, comprising sham and days 1, 3, and 7 after MCAO, were processed using Seurat in R. Seurat objects were generated for each sample with genes detected in at least three cells and cells containing at least 200 detected genes. After merging, cells with 300–7,000 detected genes and mitochondrial transcript content below 15% were retained, yielding 57,195 cells. Data were normalized using LogNormalize with a scale factor of 10,000, and 3,000 variable features were selected using the variance-stabilizing transformation method. The variable features were scaled and subjected to principal component analysis. The first 30 principal components were used for neighbor detection, clustering at a resolution of 0.5, and UMAP visualization. Cluster-enriched genes were identified using the Seurat FindAllMarkers function, and cell types were manually annotated based on these genes and established lineage markers. Feature plots were used to visualize Cxcl16 and Cxcr6 expression, and a dot plot was additionally generated for Cxcl16. Because only one sample was available at each time point, no statistical comparisons of temporal changes were performed.

### Statistical analysis

Results are reported as the mean ± SD. Statistical significance was defined as *P* < 0.05. One-way or two-way ANOVA followed by Tukey’s post hoc test was used for comparisons among three or more groups. Data were analyzed using two-tailed paired Student’s t-test for comparisons between normoxia and OGD/R conditions within each independent experiment. Statistical analyses and graph preparation were conducted with Prism 10 (GraphPad Software, USA).

## Results

### CXCL16 is upregulated at day 3 after MCAO, with Cxcl16 transcripts enriched across multiple brain cell populations

**Fig. 1:**
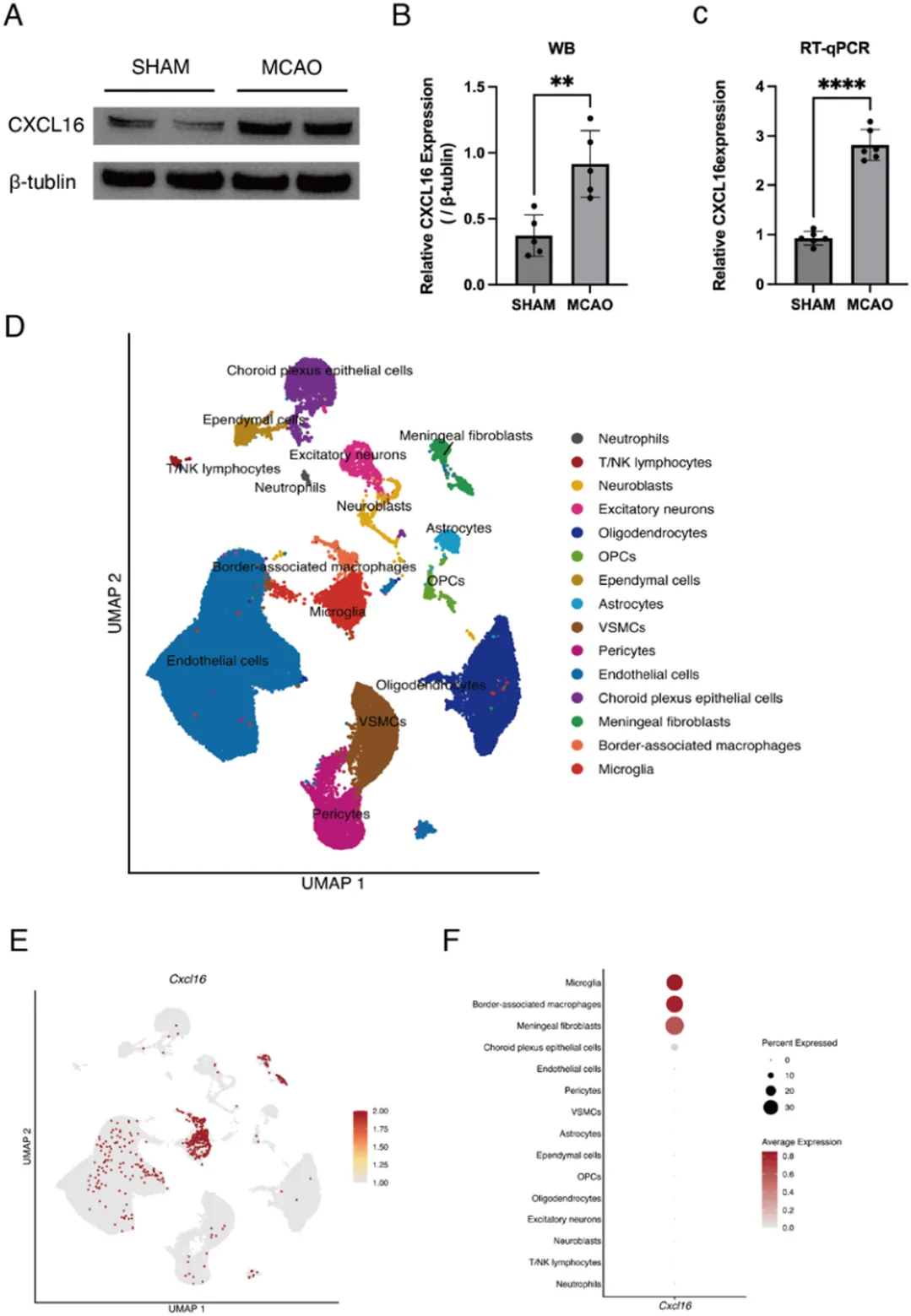
CXCL16 upregulation after cerebral ischemia and the cellular distribution of Cxcl16 transcripts. **(A-B)** Western blot analysis of CXCL16 protein expression at day 3 after MCAO in SHAM and MCAO mice (*n* = 5). **(C)** RT-qPCR analysis of CXCL16 mRNA levels (*n* = 6). **(D)** UMAP visualization of major cell populations identified in the GSE227651 single-cell RNA-sequencing dataset. **(E)** Feature plot showing the distribution of CXCL16 expression. **(F)** Dot plot showing CXCL16 expression across major cell types. **p < 0.01, ****p < 0.0001. Abbreviations: OPCs, oligodendrocyte precursor cells; VSMCs, vascular smooth muscle cells; T/NK, T and natural killer lymphocytes.

To determine whether CXCL16 may be involved in the pathological process of cerebral ischemia, we examined its expression at day 3 post-MCAO, a time point associated with substantial brain edema^19,20^. Both Western blotting (Fig. 1A-B) and RT-qPCR (Fig. 1C) revealed markedly elevated CXCL16 levels at this time point compared to sham controls. To further characterize its cellular distribution, we reanalyzed the publicly available GSE227651 single-cell RNA-sequencing dataset. CXCL16 transcripts were enriched primarily in microglia, border-associated macrophages, and meningeal fibroblast populations, suggesting that these populations may represent potential cellular sources of CXCL16 after ischemic injury (Fig. 1D–F). Analysis of receptor distribution showed that Cxcr6 transcripts were predominantly detected in T/NK lymphocytes, whereas no detectable Cxcr6 expression was observed in the annotated microglial population (Supplementary Fig. 2G).

### rCXCL16 modulates inflammation- and repair-associated gene expression in primary microglia under OGD/R conditions

Microglia are central regulators of neuroinflammation following cerebral ischemia and express the CXCL16 receptor CXCR6^8,21^. Given that CXCL16 was elevated at day 3 post-MCAO, we investigated whether it modulates microglial functional states. Primary microglia were isolated and characterized by immunofluorescence staining for Iba-1 and CD206 (Supplementary Fig. 2 A). Double immunofluorescence staining further demonstrated CXCR6 immunoreactivity in Iba1-positive cells under basal conditions (Supplementary Fig. 2B).

The OGD/R model used for primary microglia was optimized as follows. Cell viability was assessed after five different durations of OGD followed by 24 h of reoxygenation. Four hours of OGD reduced microglial viability to approximately 50% of the normoxic control level and was therefore selected for subsequent experiments (Supplementary Fig. 2 C). Using this condition, we examined the endogenous expression of both components of the CXCL16–CXCR6 axis. Western blotting showed that CXCL16 and CXCR6 were detectable in primary microglia under normoxic conditions and that the expression of both proteins was significantly increased following OGD/R (Supplementary Fig. 2D–F). These findings indicate that cultured primary microglia express both CXCL16 and CXCR6 under the experimental conditions used and may represent a potential source of CXCL16 under OGD/R conditions.

**Fig. 2:**
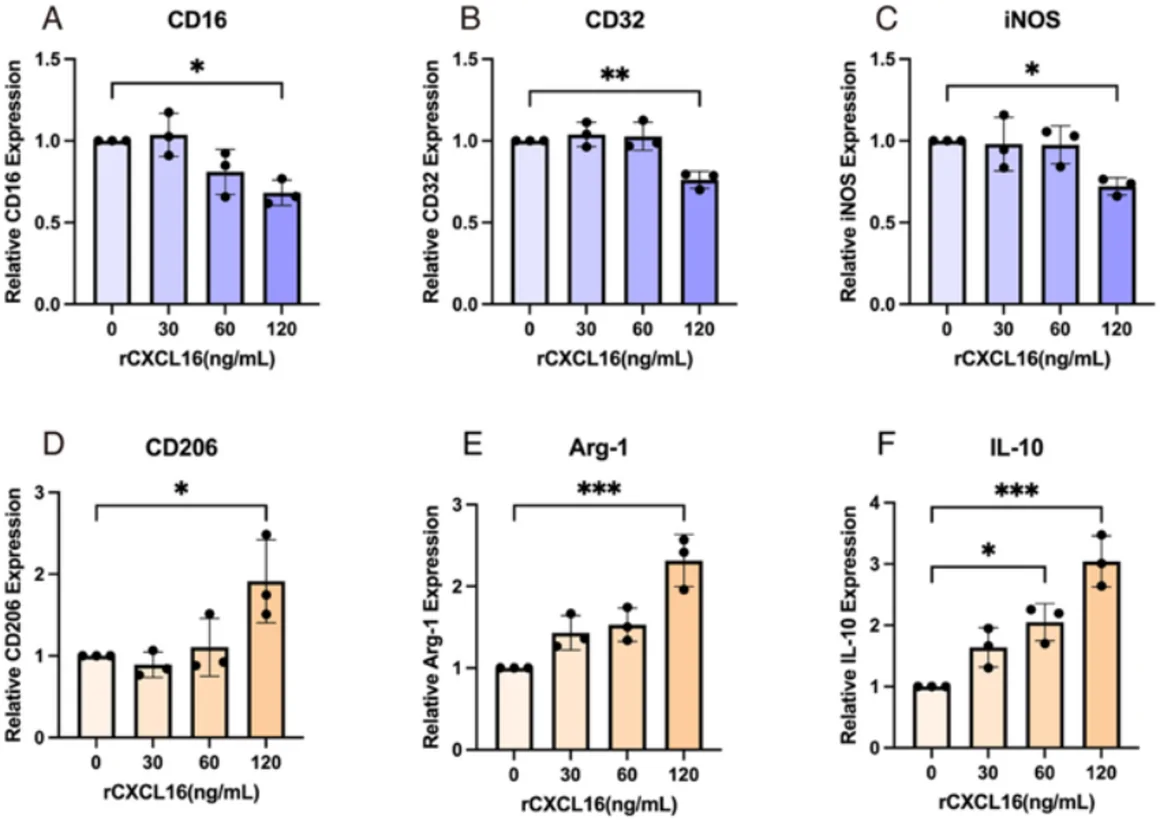
Effects of rCXCL16 on inflammation- and repair-associated gene expression in primary microglia under OGD/R conditions. RT-qPCR analysis of inflammation-associated markers, including CD16, CD32, and iNOS (A–C), and repair-associated markers, including CD206, Arg-1, and IL-10 (D–F), in primary microglia exposed to OGD/R and treated with the indicated concentrations of rCXCL16 (*n* = 3). *p < 0.05, **p < 0.01, ***p < 0.001.

To examine whether rCXCL16 alters microglial responses under OGD/R conditions, we assessed the expression of inflammation-associated markers, including CD16, CD32, and iNOS, together with repair-associated markers, including CD206, Arg-1, and IL-10. Treatment with 120 ng/mL rCXCL16 significantly reduced the expression of the inflammation-associated markers CD16, CD32, and iNOS following OGD/R (Fig. 2A-C). In parallel, rCXCL16 increased the expression of the repair-associated markers CD206 and Arg-1 (Fig. 2D and E), while IL-10 expression was increased at both 60 and 120 ng/mL (Fig. 2F). These findings demonstrate that rCXCL16 modulates selected inflammation- and repair-associated genes in primary microglia under OGD/R conditions (see Fig. 2).

### rCXCL16 partially reverses LPS-induced changes in inflammation- and repair-associated gene expression of primary microglia

**Fig. 3:**
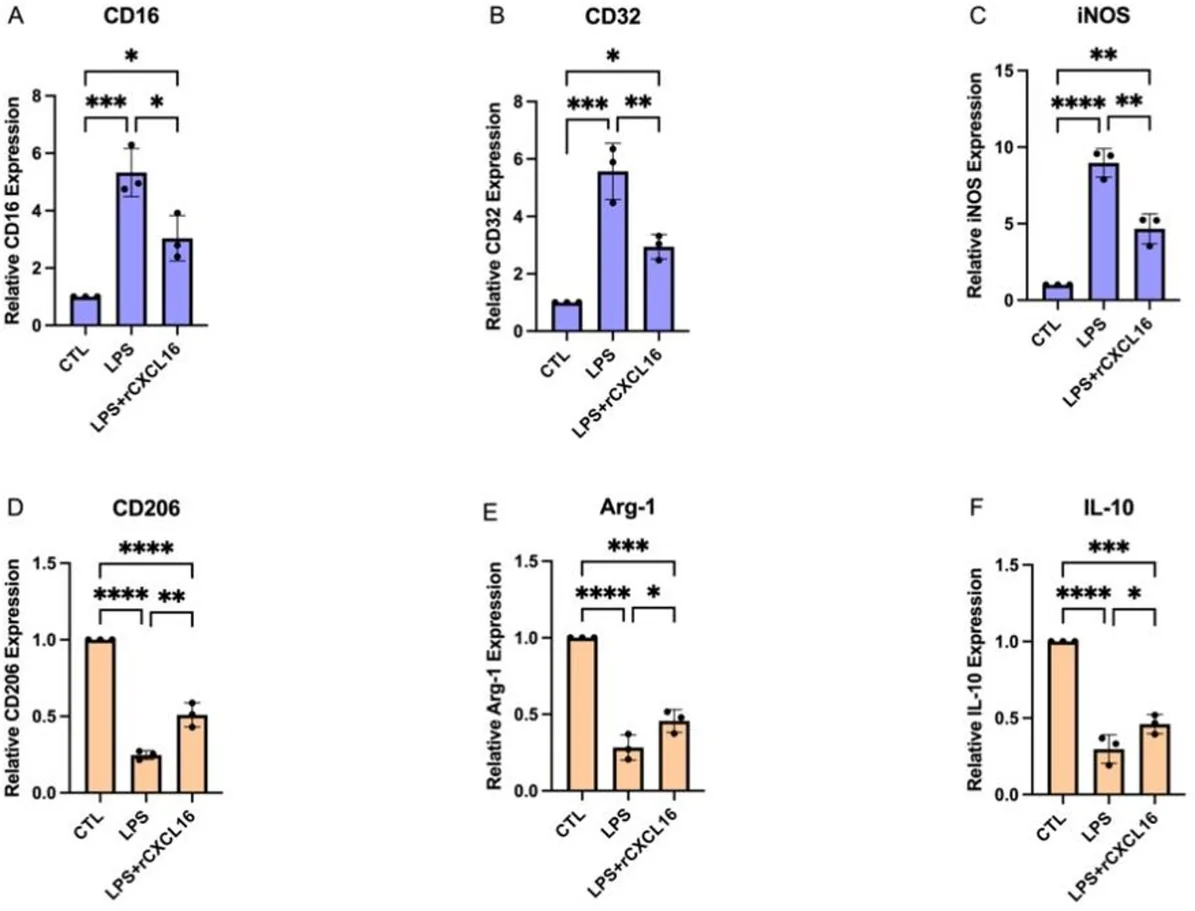
Effects of rCXCL16 on inflammation- and repair-associated gene expression in LPS-stimulated primary microglia. RT-qPCR analysis of selected inflammation-associated markers, including CD16, CD32, and iNOS **(A–C)**, and repair-associated markers, including CD206, Arg-1, and IL-10 **(D–F)**, in Control, LPS, and LPS + rCXCL16 (120 ng/mL) groups (*n* = 3). *p < 0.05, **p < 0.01, ***p < 0.001, ****p < 0.0001.

We next examined whether the modulatory effects of rCXCL16 were also observed in response to a non-ischemic inflammatory stimulus. LPS stimulation significantly increased the expression of CD16, CD32, and iNOS and reduced the expression of CD206, Arg-1, and IL-10 compared with the control group (Fig. 3A–F). rCXCL16 treatment significantly attenuated these LPS-induced changes. Nevertheless, all six markers remained significantly different from control levels, indicating that rCXCL16 partially, but not completely, reversed the transcriptional response induced by LPS.

### rCXCL16-pretreated primary microglia enhance HT-22 cell viability in a co-culture model under OGD/R conditions

**Fig. 4:**
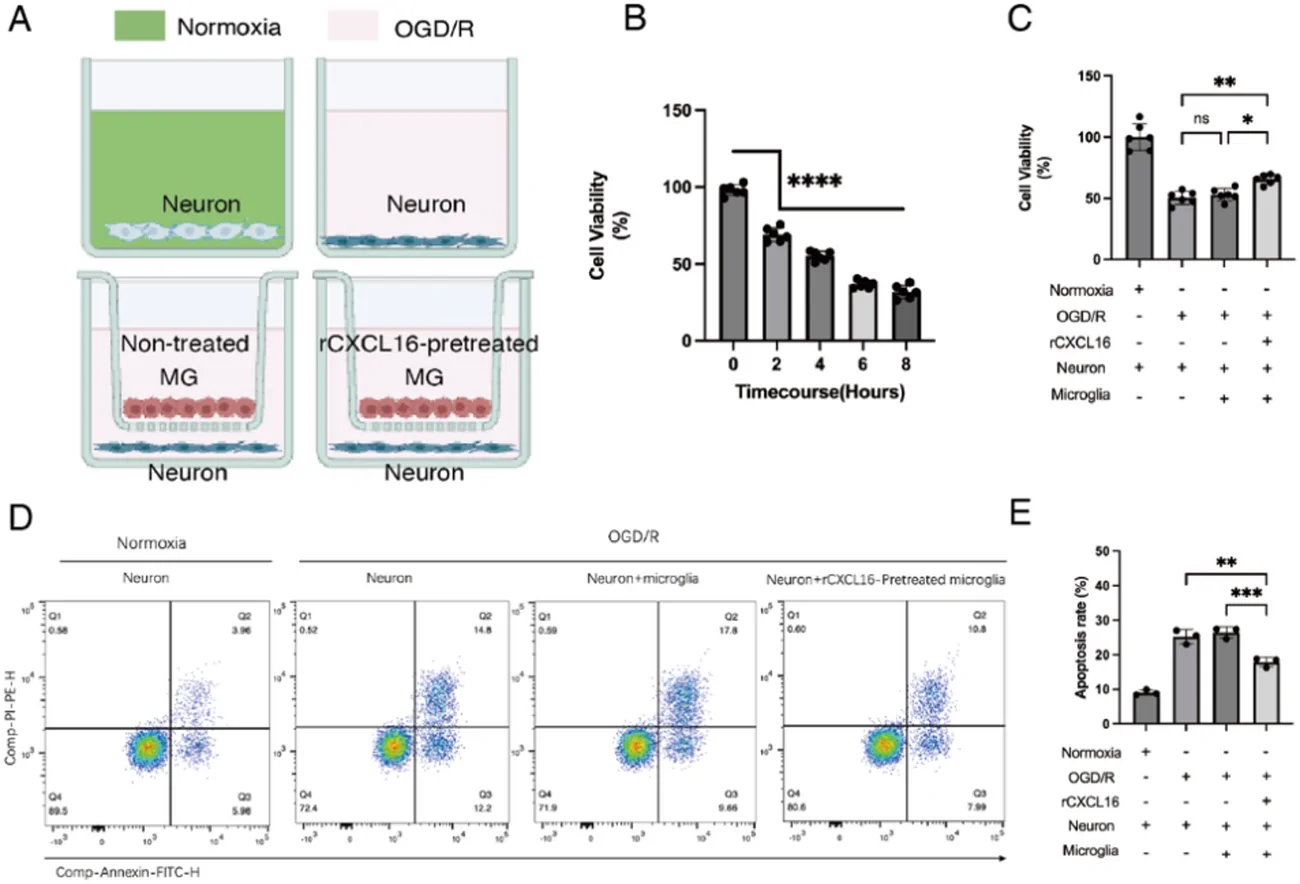
rCXCL16-pretreated microglia protect HT-22 cells against OGD/R-induced viability loss and apoptosis in co-culture. **(A)** Schematic of the co-culture model. MG, microglia. **(B)** MTT assay determining the optimal OGD duration in HT-22 cells. Co-culture with rCXCL16-pretreated microglia significantly improved neuronal viability **(C)** and reduced neuronal apoptosis **(E) **following OGD/R exposure. Representative FACS plots **(D)** show neuronal apoptosis in each group under OGD/R conditions. Data in **(B)** and **(C)** are from six technical replicates, typical results from three independent replicates. Data in **(E)** are from three independent experiments (each point represents one experiment). ns, not significant; *p < 0.05, **p < 0.01, ***p < 0.001, ****p < 0.0001.

Given the effects of rCXCL16 on inflammation- and repair-associated gene expression in microglia under both LPS and OGD/R conditions, we next asked whether these changes were associated with neuronal protection. A co-culture model of primary microglia and HT-22 neurons was therefore established to evaluate the protective potential of rCXCL16-pretreated microglia under OGD/R conditions (Fig. 4A). An MTT assay was first performed in HT-22 cells to determine the optimal duration of OGD, and we chose 4 h for further assays (Fig. 4B). The HT-22 cells were then co-cultured with microglia during the reoxygenation phase. Under OGD/R conditions, rCXCL16-pretreated microglia markedly enhanced neuronal viability relative to neuron-only cultures or co-cultures with non-treated microglia (Fig. 4C). Given that apoptosis is the predominant mode of cell death within the ischemic penumbra, we performed flow cytometry (FACS) to quantify neuronal apoptosis (Fig. 4D). Consistently, under OGD/R conditions, the apoptotic rate was markedly decreased in the presence of rCXCL16-treated microglia versus both the neuron-only group and the co-culture group with untreated microglia (Fig. 4E).

Because the non-contact co-culture findings suggested the involvement of microglia-derived soluble factors, we performed complementary experiments to determine whether the protective effect could be reproduced using microglial conditioned medium and whether rCXCL16 acted directly on HT-22 cells. Direct treatment with rCXCL16 at 60, 120, or 240 ng/mL did not significantly improve HT-22 cell viability following OGD/R (Supplementary Fig. 2H). In contrast, conditioned medium derived from rCXCL16-treated microglia significantly increased HT-22 cell viability compared with conditioned medium from vehicle-treated microglia, whereas vehicle-treated microglial conditioned medium did not differ significantly from the OGD/R group (Supplementary Fig. 2I). These findings support the involvement of soluble factors released by rCXCL16-treated microglia rather than a prominent direct protective effect of rCXCL16 on HT-22 cells.

### rCXCL16 attenuates infarct volume and enhances neurological outcomes following MCAO

**Fig. 5:**
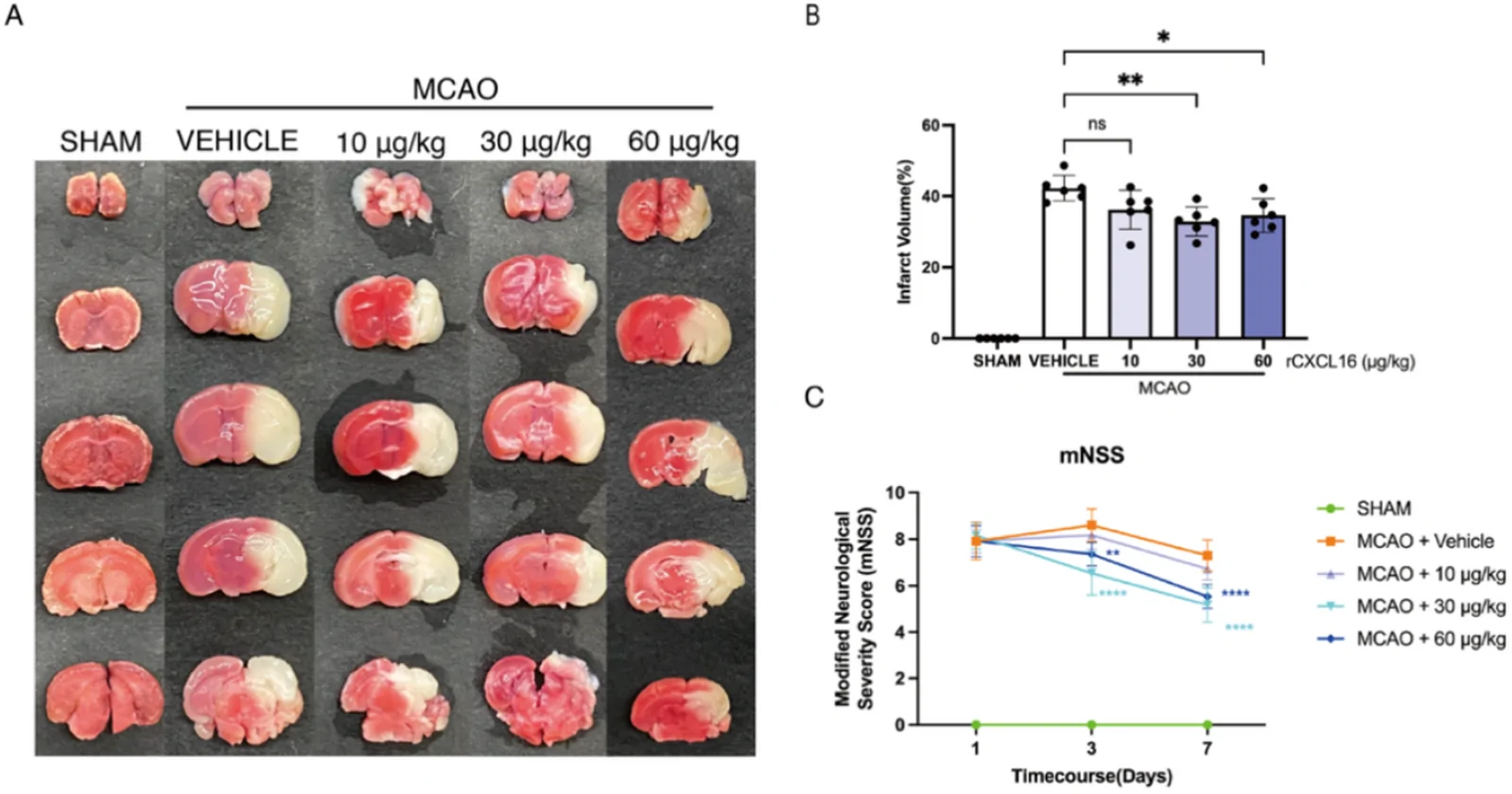
rCXCL16 reduces infarct volume and neurological deficits in MCAO mice. Mice were treated with vehicle or rCXCL16 (10, 30, or 60 µg/kg). At 24 h post-MCAO, TTC staining **(A-B)** showed that rCXCL16 at 30 and 60 µg/kg significantly reduced infarct volume versus vehicle, with a bell-shaped dose–response and maximal effect at 30 µg/kg (*n* = 6 mice per group). Neurological scores **(C)** showed parallel improvements (*n* = 10–12 mice per group). ns, not significant; *p < 0.05, **p < 0.01, ****p < 0.0001.

To evaluate its neuroprotective effects in vivo, rCXCL16 was administered intracerebroventricularly at doses of 10, 30, or 60 µg/kg at 1 h after MCAO. TTC staining revealed that treatment with rCXCL16 at doses of 30 and 60 µg/kg, but not 10 µg/kg, markedly decreased infarct volume compared with the vehicle group. Notably, this effect exhibited a bell-shaped dose–response pattern, with the intermediate dose showing the most pronounced reduction (Fig. 5A-B). Consistent with the TTC results, neurological function was also significantly improved in rCXCL16-treated mice, with the greatest improvement observed at the intermediate dose (Fig. 5C). We therefore chose 30 µg/kg as the optimal dose for further in vivo testing.

### rCXCL16 alters inflammation- and repair-associated marker profiles in microglia/macrophages after MCAO

**Fig. 6:**
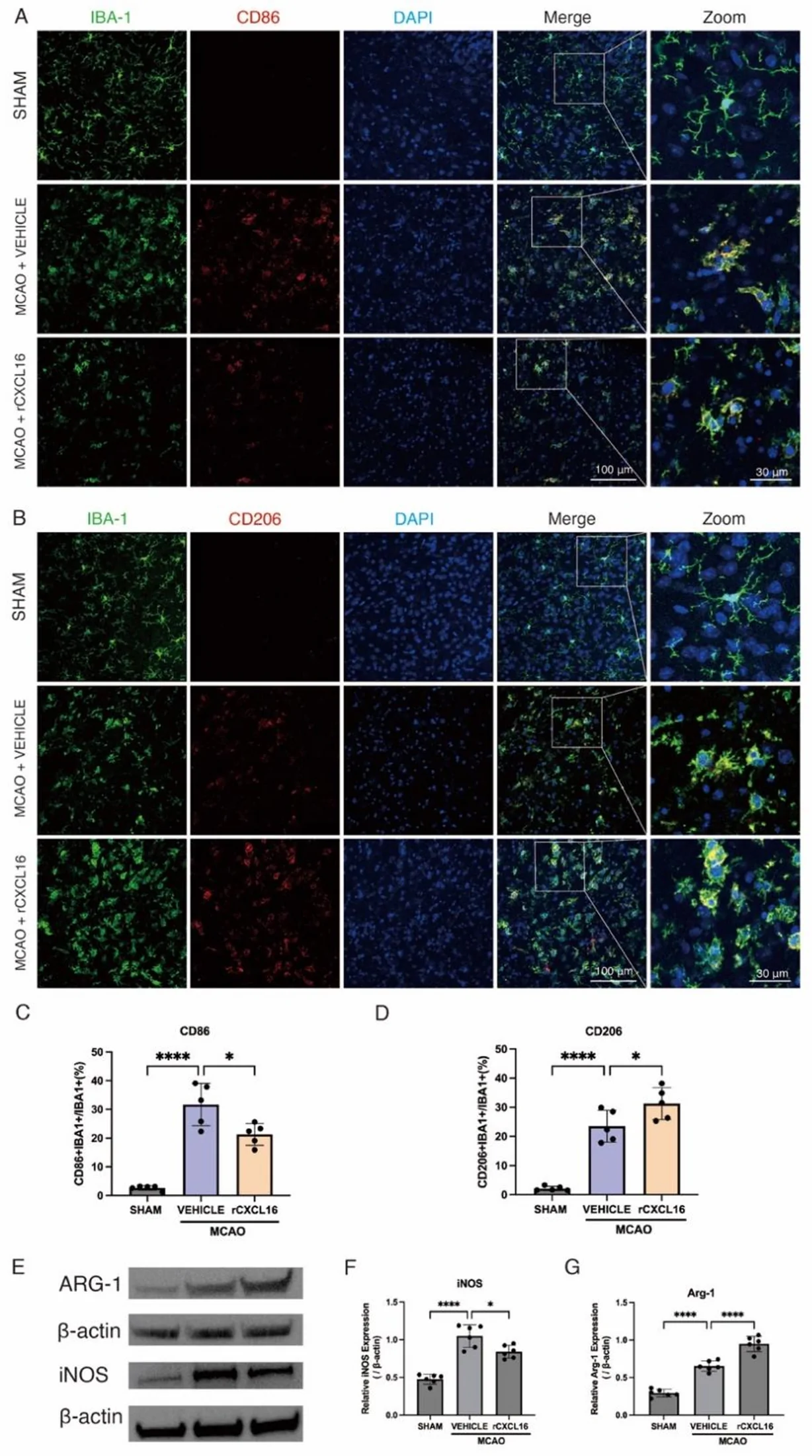
rCXCL16 modulates inflammation- and repair-associated marker profiles after MCAO. Immunofluorescence analysis showed that rCXCL16 reduced CD86^+^/Iba-1^+^ microglia/macrophages and increased CD206^+^/Iba-1^+^ microglia/macrophages in the peri-infarct region (*n* = 5). Western blotting showed reduced iNOS and increased Arg-1 protein expression in ischemic brain tissue (*n* = 6). Scale bar = 100 μm. *p < 0.05, ****p < 0.0001.

To examine whether rCXCL16 altered microglia/macrophage-associated responses in vivo, we assessed CD86 and CD206 immunoreactivity in Iba-1^+^ cells, together with iNOS and Arg-1 protein expression in ischemic brain tissue. MCAO increased the number of CD86^+^/Iba-1^+^ cells in the peri-infarct region (Fig. 6A and C), whereas rCXCL16 treatment reduced this population and increased the number of CD206^+^/Iba-1^+^ cells (Fig. 6B and D). Consistently, rCXCL16 reduced iNOS protein expression and increased Arg-1 expression in ischemic brain tissue (Fig. 6E-G). These findings indicate that rCXCL16 treatment is associated with a shift toward reduced inflammatory and enhanced repair-associated marker expression after MCAO (see Fig. 6).

### rCXCL16 reduces apoptosis in vivo

**Fig. 7:**
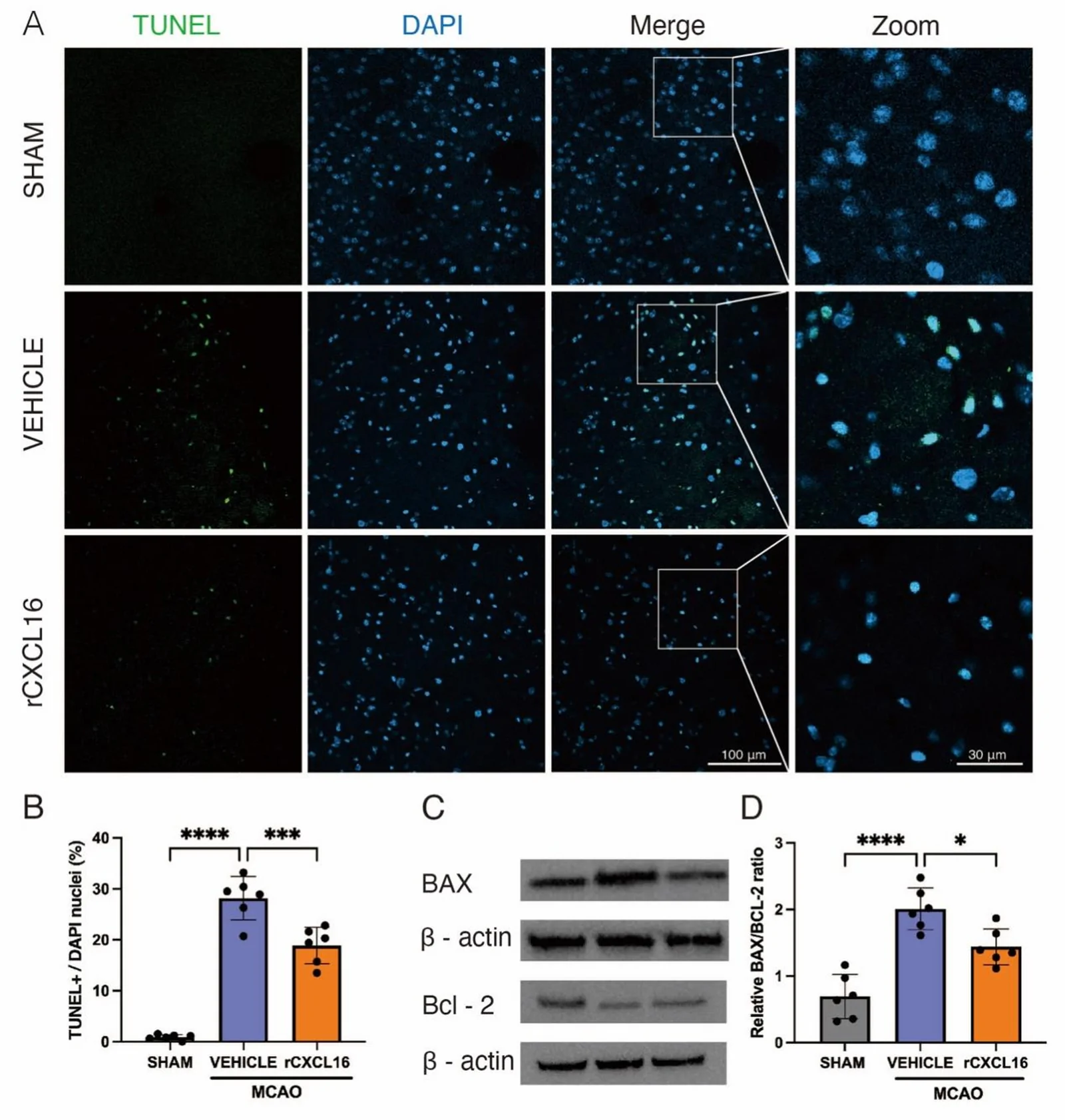
rCXCL16 reduces apoptosis in the ischemic cortex following MCAO. TUNEL staining **(A-B)** and Western blot analysis of Bax and Bcl-2 **(C, D)** showed that rCXCL16 treatment substantially decreased apoptotic cell numbers and enhanced the Bcl-2/Bax ratio (*n* = 6). *p < 0.05, ***p < 0.001, ****p < 0.0001.

To determine whether rCXCL16 treatment attenuates apoptotic cell death, we next examined cell apoptosis in the ischemic cortex. TUNEL staining displayed an elevation of apoptotic cells in the vehicle-treated MCAO group. However, rCXCL16 administration markedly lowered the count of TUNEL-positive cells (Fig. 7A-B). Western blotting was further performed to confirm anti-apoptotic effects. Compared to the vehicle group, rCXCL16 significantly downregulated Bax expression and up-regulated Bcl-2 levels, leading to a markedly reduced Bax/Bcl-2 ratio (Fig. 7C-D). These data demonstrate that rCXCL16 effectively suppresses apoptosis, contributing to its neuroprotective role in ischemic stroke.

## Discussion

Chemokines have traditionally been characterized by their ability to recruit immune cells to sites of injury or inflammation, and extensive studies have focused on their roles in leukocyte trafficking under various pathological conditions^22^. However, emerging evidence suggests that chemokines can also directly regulate cellular states within the central nervous system^23,24^, raising the possibility that exogenously administered chemokines may hold therapeutic potential in central nervous system disorders.

This study examined whether rCXCL16 modulates microglial responses and ischemic injury. In primary microglia, rCXCL16 reduced inflammation-associated and increased repair-associated gene expression under OGD/R and LPS stimulation. After MCAO, rCXCL16 reduced TTC-defined infarct volume and mNSS scores, decreased CD86 and increased CD206 immunoreactivity in IBA1 + microglia/macrophages, and lowered iNOS while elevating Arg-1 in ischemic brain tissue. rCXCL16-treated microglia increased HT-22 viability and reduced apoptosis in co-culture; their conditioned medium reproduced the viability benefit, whereas direct rCXCL16 did not. Together, these findings associate rCXCL16-mediated microglial modulation with reduced ischemic injury and improved neurological outcomes.

Although scRNA-seq identified several CXCL16-expressing cell populations, their relative contributions to the post-ischemic increase in tissue CXCL16 remain unresolved. Furthermore, transcript detection does not establish CXCL16 protein production or release in its soluble form, which will require spatial and protein-level validation.

The cellular distribution of CXCR6 also warrants cautious interpretation. In GSE227651, Cxcr6 transcripts were detected predominantly in T/NK lymphocytes and were not detected in the annotated microglial population, whereas sporadic transcript detection was observed in the larger integrated MCAO atlas SCP3078^25^. Such variation may reflect low transcript abundance, sequencing dropout, differences in cellular composition or annotation, or biological heterogeneity among datasets. Although we confirmed CXCR6 protein expression in cultured primary microglia and observed responses to rCXCL16 in vitro, neonatal cultured microglia may not fully reproduce the receptor expression profile of adult microglia in the ischemic brain. Moreover, CXCR6 dependence was not examined using pharmacological blockade or genetic deletion. Thus, the in vivo effects of rCXCL16 cannot be attributed exclusively to microglia and may also involve infiltrating CXCR6-expressing immune populations, particularly T/NK lymphocytes. Tissue-level colocalization and cell-specific inhibition or deletion of CXCR6 will be required to define the relative contribution of these populations.

Microglial responses after ischemic stroke are temporally dynamic and highly heterogeneous and cannot be adequately represented by a binary M1/M2 classification^26^. Individual microglia may simultaneously exhibit inflammatory, homeostatic, phagocytic, metabolic, and repair-associated features, depending on the stage of injury, anatomical location, and local cellular environment. Therefore, CD16, CD32, iNOS, CD206, Arg-1, and IL-10 should be interpreted as selected state-associated markers rather than definitive indicators of discrete and mutually exclusive M1 or M2 phenotypes. In the present study, rCXCL16 reduced selected inflammation-associated markers and enhanced selected repair-associated markers under both OGD/R and LPS stimulation. These findings indicate modulation of the microglial transcriptional profile but do not establish conversion from one discrete state to another. In addition, our in vitro experiments used neonatal primary microglia, whose transcriptional and functional states may differ from those of adult microglia and may be further altered by isolation and culture^27–29^. Therefore, the present in vitro findings should not be directly extrapolated to adult microglial responses in the ischemic brain.

Building upon the observed changes in inflammation- and repair-associated gene expression, we next examined whether rCXCL16-treated microglia influenced HT-22 cell survival under OGD/R conditions. In the non-contact co-culture system, untreated microglia did not significantly improve HT-22 cell viability, whereas rCXCL16-pretreated microglia increased cell viability and reduced apoptosis. Further supporting the co-culture findings, conditioned medium from rCXCL16-treated microglia significantly improved HT-22 cell viability, whereas direct rCXCL16 treatment had no significant protective effect across the tested concentration range. Together, these findings support the involvement of soluble factors released by rCXCL16-treated microglia, rather than a prominent direct effect of rCXCL16 on HT-22 cells. However, the specific soluble mediators involved and their dependence on microglial CXCR6 signaling remain to be determined.

In vivo, rCXCL16 treatment was associated with reduced ischemic injury, improved neurological outcomes, and altered inflammation- and repair-associated marker profiles after MCAO. However, because IBA1 does not distinguish resident microglia from infiltrating macrophages and iNOS and Arg-1 were measured in ischemic brain tissue homogenates, these changes cannot be attributed specifically to microglia. Accordingly, the in vivo findings support an association between rCXCL16 treatment and modulation of post-ischemic inflammatory responses rather than establishing a microglia-specific causal mechanism.

Together, these results indicate that rCXCL16 modulates microglial responses and is associated with reduced neuronal injury in vitro and improved outcomes after MCAO.

This study has several limitations. Although the conditioned-medium experiments support the involvement of microglia-derived soluble factors, the responsible mediators remain unidentified. CXCR6 expression was confirmed in cultured primary microglia, but receptor dependence and downstream signaling were not examined using pharmacological blockade or genetic manipulation, and contributions from other CXCR6-expressing cells in vivo cannot be excluded. The cellular specificity and causal contribution of microglia in vivo also remain unresolved because IBA1 does not distinguish resident microglia from infiltrating macrophages, iNOS and Arg-1 were measured in tissue homogenates, and TUNEL staining was not combined with lineage-specific markers. Furthermore, cultured neonatal microglia may not fully recapitulate adult microglial states, and the lack of biological replication at each time point in the single-cell dataset precluded temporal inference and quantitative attribution of CXCL16 sources. Future studies using cell-type-specific CXCR6 manipulation, adult microglial models, and spatial or protein-level analyses are required.

## Supplementary Information

Below is the link to the electronic supplementary material.

Supplementary Material 1

Supplementary Material 2

Supplementary Material 3

## Data Availability

Data are available from the corresponding author on request. The single-cell RNA-sequencing data analyzed in this study are publicly available in the Gene Expression Omnibus under accession number GSE227651.
