# Supplementary material for: Recombinant CXCL16 reduces brain injury by modulating microglial phenotype and attenuating apoptosis in acute ischemic stroke
Source: Sci Rep. 2026 Aug 22;16:26358. doi: 10.1038/s41598-026-66143-7 (PMC13499807; doi:10.1038/s41598-026-66143-7)
Supplement: Supplementary file 1 [file 41598_2026_66143_MOESM1_ESM.pdf]

Supplementary Table 1: Primers for RT-qPCR analysis

CD16	Forward: TTTGGACACCCAGATGTTTCAG<br><br>Reverse: GTCTTCCTTGAGCACCTGGATC
CD32	Forward: AATCCTGCCGTTCCTACTGATC<br><br>Reverse: GTGTCACCGTGTCTTCCTTGAG
iNOS	Forward: CAAGCACCTTGGAAGAGGAG<br><br>Reverse: AAGGCCAAACACAGCATACC
CD206	Forward: CAAGGAAGGTTGGCATTTGT<br><br>Reverse: CCTTTCAGTCCTTTGCAAGC
Arg-1	Forward: TCACCTGAGCTTTGATGTCG<br><br>Reverse: CTGAAAGGAGCCCTGTCTTG
IL-10	Forward: CCAAGCCTTATCGGAAATGA<br><br>Reverse: TTTTCACAGGGGAGAAATCG
β-actin	Forward: GGCTGTATTCCCCTCCATCG<br><br>Reverse: CCAGTTGGTAACAATGCCATGT

Primer sequences used to amplify mouse CD16, CD32, iNOS, CD206, Arg-1, IL-10, and β-actin. β-actin served as the internal control.
