# Supplementary material for: Recombinant CXCL16 reduces brain injury by modulating microglial phenotype and attenuating apoptosis in acute ischemic stroke
Source: Sci Rep. 2026 Aug 22;16:26358. doi: 10.1038/s41598-026-66143-7 (PMC13499807; doi:10.1038/s41598-026-66143-7)
Supplement: Supplementary file 2 [file 41598_2026_66143_MOESM2_ESM.pdf]

Fig. 1A

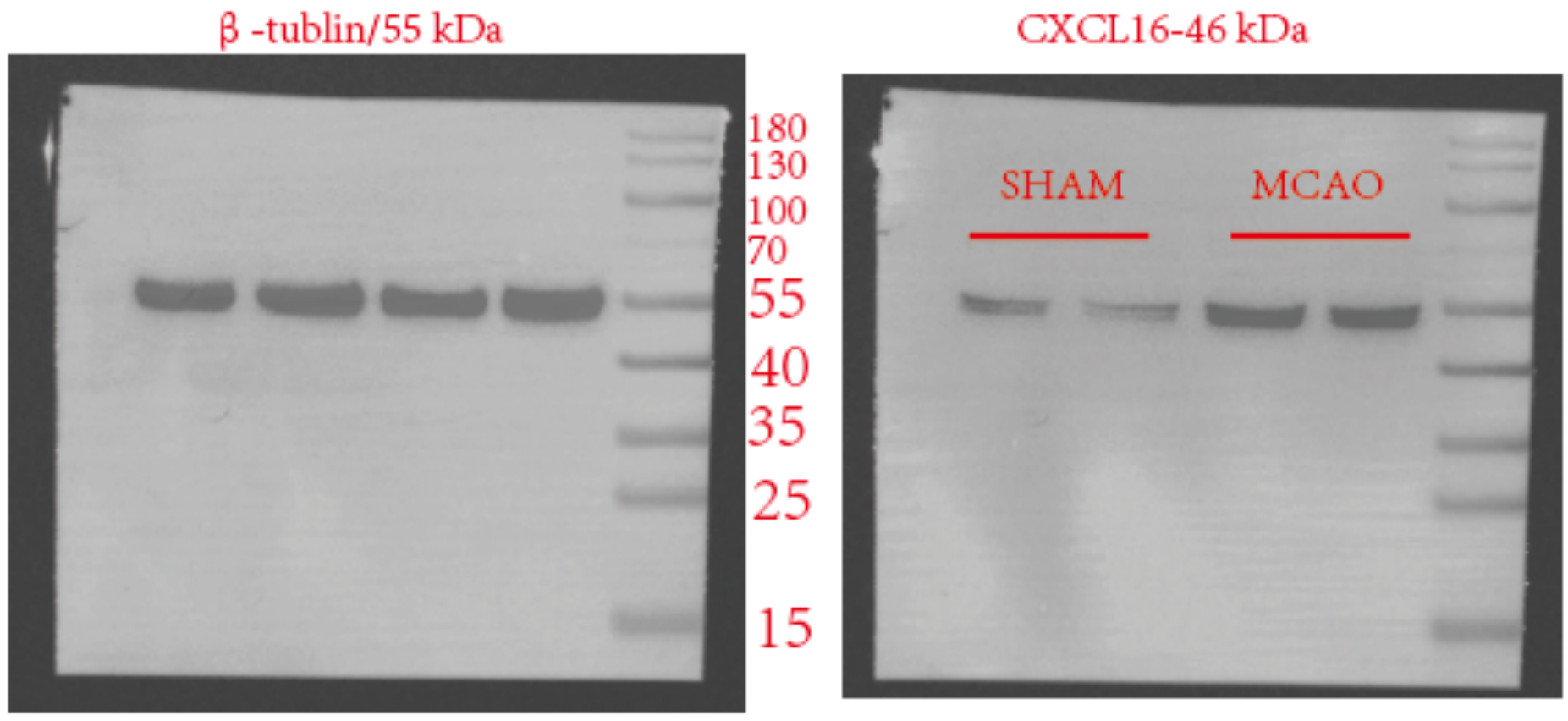

Fig. 6E

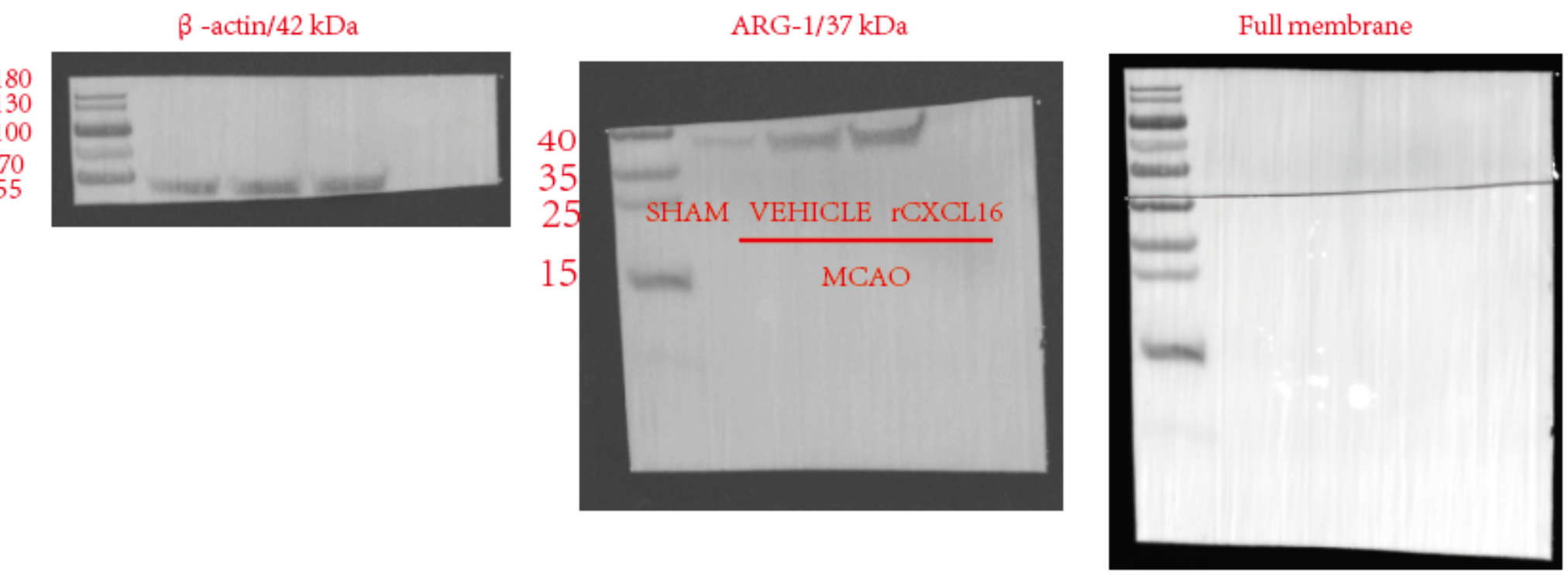

Fig. 6E

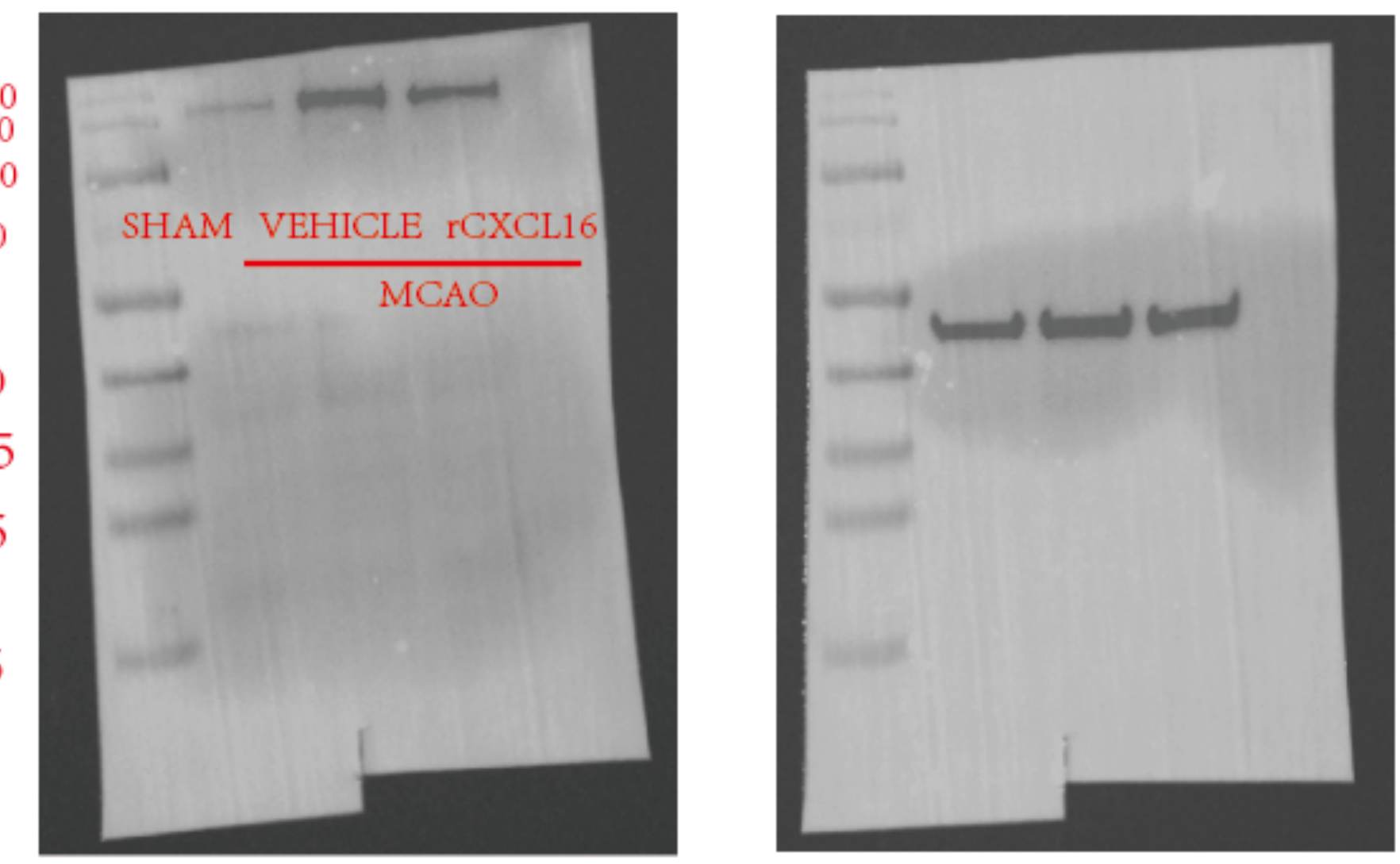

Fig. 7C

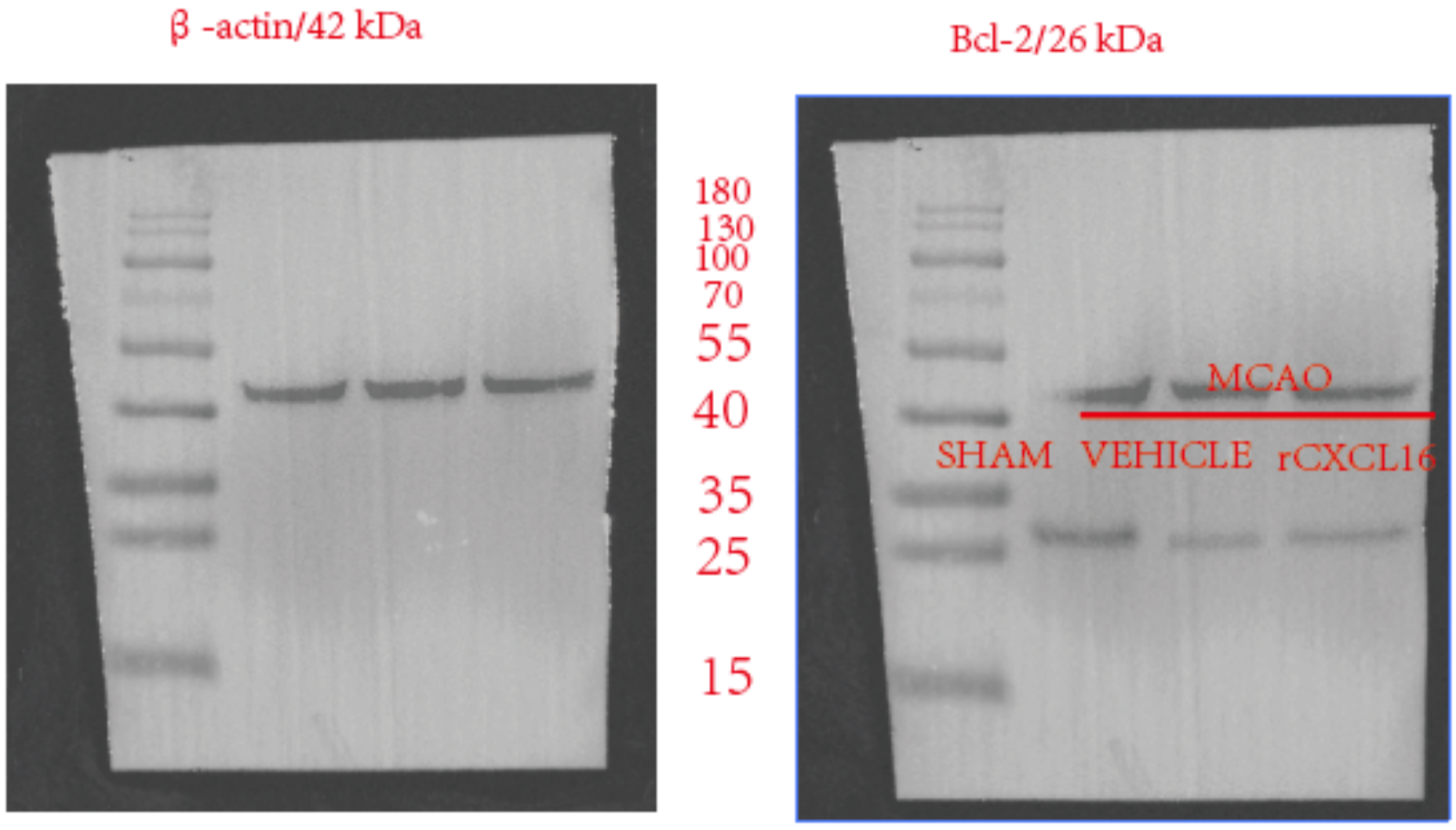
β -actin/42 kDa
Bcl-2/26 kDa
180
130
100
70
55
40
35
25
15
MCAO
SHAM
VEHICLE
rCXCL16

Fig. 7C

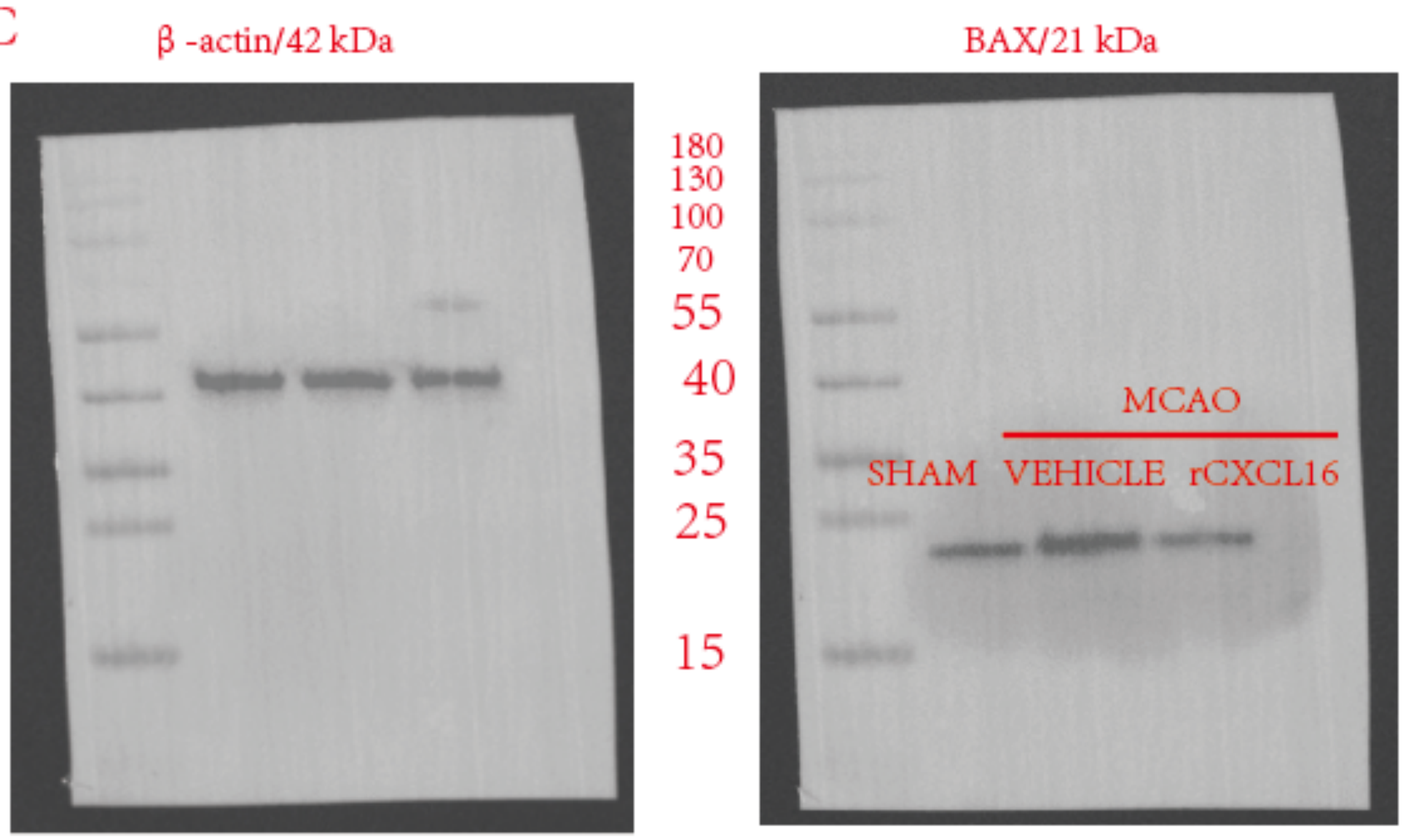
β -actin/42 kDa
BAX/21 kDa
180
130
100
70
55
40
35
25
15
MCAO
SHAM
VEHICLE
rCXCL16
