# Supplementary material for: Recombinant CXCL16 reduces brain injury by modulating microglial phenotype and attenuating apoptosis in acute ischemic stroke
Source: Sci Rep. 2026 Aug 22;16:26358. doi: 10.1038/s41598-026-66143-7 (PMC13499807; doi:10.1038/s41598-026-66143-7)
Supplement: Supplementary file 3 [file 41598_2026_66143_MOESM3_ESM.pdf]

Supplementary Figure 2

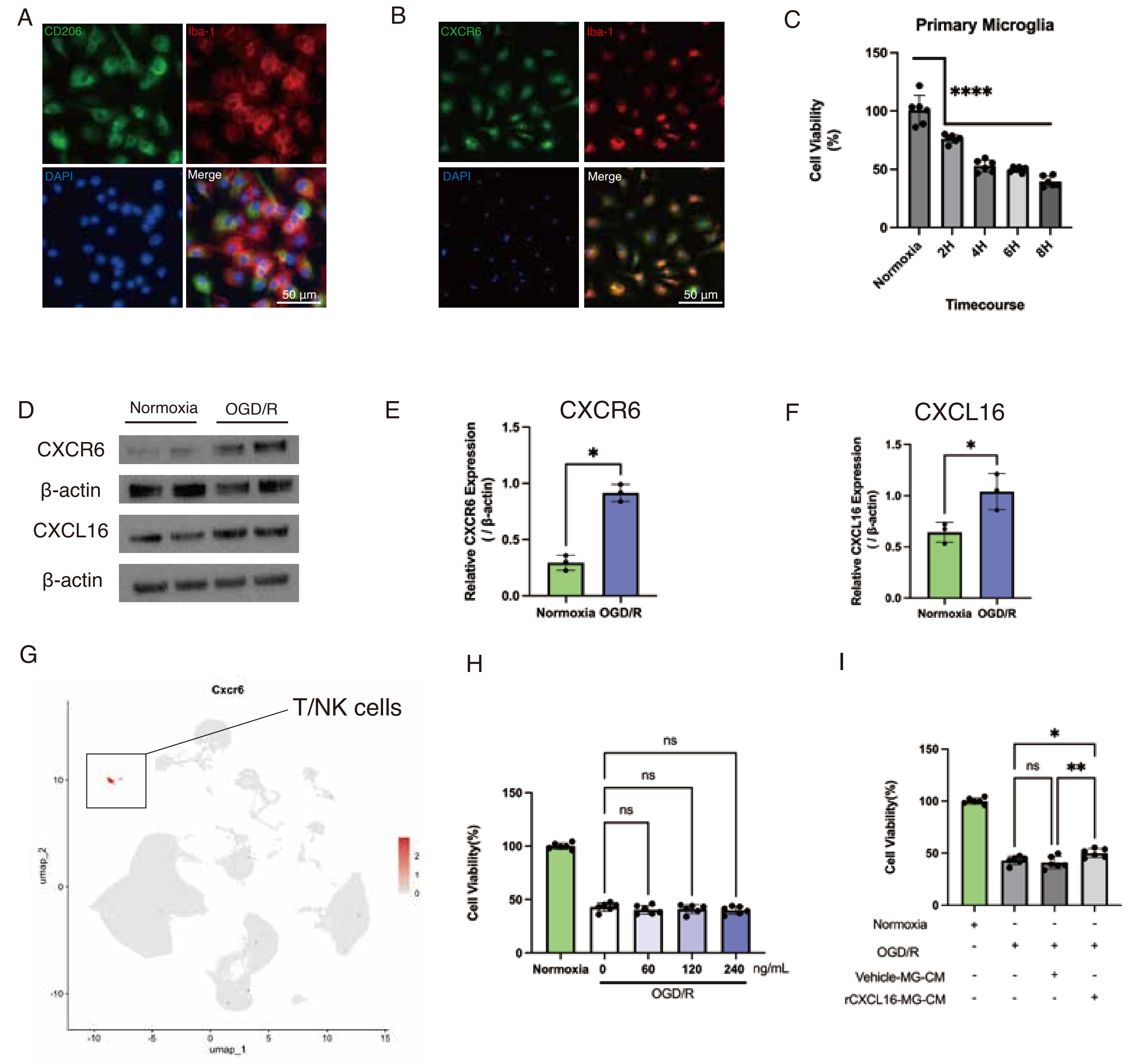

Supplementary Figure 2.
(A) Immunofluorescence characterization of primary microglial cultures using Iba-1 and CD206.
(B) Representative double immunofluorescence staining showing CXCR6 immunoreactivity in Iba-1-positive primary microglia under basal culture conditions.
(C) MTT assay assessing primary microglial viability after different durations of OGD followed by 24 h of reoxygenation. Four hours of OGD was selected for subsequent experiments.
(D and F) Western blot analysis and quantification of CXCR6 and CXCL16 protein expression in primary microglia under normoxic and OGD/R conditions (n=3).
(G) Feature plot of Cxcr6 expression across cell clusters, showing predominant expression in T/NK cells.
(H) MTT analysis of HT-22 cell viability following direct treatment with rCXCL16.
(I) MTT analysis of HT-22 cell viability following exposure to microglia-conditioned medium.
Data in (C), (H) and (I) are from six technical replicates, typical results from three independent replicates.
Data in (E) and (F) are from three independent experiments (each point represents one experiment).
ns, not significant; *$p < 0.05$; **$p < 0.01$; ****$p < 0.0001$.
